# The Effect of Transcription Factor *MYB14* on Defense Mechanisms in *Vitis quinquangularis*-Pingyi

**DOI:** 10.3390/ijms21030706

**Published:** 2020-01-21

**Authors:** Yangyang Luo, Qingyang Wang, Ru Bai, Ruixiang Li, Lu Chen, Yifan Xu, Ming Zhang, Dong Duan

**Affiliations:** Key Laboratory of Resource Biology and Biotechnology in Western China, Ministry of Education, College of Life Sciences, Northwest University, Xi’an 710069, Shaanxi, China; yyl928781001@gmail.com (Y.L.); qingyangwang058@gmail.com (Q.W.); bairu8517@gmail.com (R.B.); liruixiang928@gmail.com (R.L.); chenluailiukang@gmail.com (L.C.); xyf15686060863@gmail.com (Y.X.); Clover20200120@gmail.com (M.Z.)

**Keywords:** Chinese wild *Vitis*, defense, ETI, *MYB14*, PTI, stilbene

## Abstract

In the current study, we identified a transcription factor, *MYB14*, from Chinese wild grape, *Vitis quinquangularis*-Pingyi (*V. quinquangularis*-PY), which could enhance the main stilbene contents and expression of stilbene biosynthesis genes (*StSy*/*RS*) by overexpression of *VqMYB14*. The promoter of *VqMYB14* (*pVqMYB14*) was shown to be induced as part of both basal immunity (also called pathogen-associated molecular pattern (PAMP)-triggered immunity, PTI) and effector-triggered immunity (ETI), triggered by the elicitors flg22 and harpin, respectively. This was demonstrated by expression of *pVqMYB14* in *Nicotiana benthamiana* and *Vitis*. We identified sequence differences, notably an 11 bp segment in *pVqMYB14* that is important for the PTI/ETI, and particularly for the harpin-induced ETI response. In addition, we showed that activation of the *MYB14* promoter correlates with differences in the expression of *MYB14* and stilbene pattern induced by flg22 and harpin. An experimental model of upstream signaling in *V. quinquangularis*-PY is presented, where early defense responses triggered by flg22 and harpin partially overlap, but where the timing and levels differ. This translates into a qualitative difference with respect to patterns of stilbene accumulation.

## 1. Introduction

To combat the many biotic stresses to which they are exposed, plants have evolved two distinct layers of innate immunity [1]. The first is a basal immunity that is activated by pathogen-associated molecular patterns (PAMPs) and is therefore named PAMP-triggered immunity (PTI) [2]. One well-characterized PAMP is bacterial flagellin-flg22, which activates early defense responses in plants, including the cellular influx of H^+^ and Ca^2+^, the production of reactive oxygen species (ROS), the activation of mitogen-activated protein kinase (MAPK) cascades and transcription factors, and phytoalexin accumulation [3,4]. During coevolution with their hosts, pathogens have evolved effectors that can block PTI, thereby allowing the pathogen to infect the host [5]. In parallel, plants have evolved resistance (R) genes, the products of which recognize the microbial effectors, leading to a hypersensitive response (HR), which represents the second layer of defense, called effector-triggered immunity (ETI) [1]. Harpin, which belongs to a group of effector proteins exported by a bacterial T3SS secretion system, is often used experimentally to mimic certain aspects of ETI and has been extensively studied for its ability to initiate hypersensitive cell death and to induce systemic acquired resistance (SAR) [6,7].

Although the PTI and ETI responses are activated in the plant by recognition of different pathogen-derived molecules, they have several signaling events in common, including calcium influx, activation of an apoplastic oxidative burst, MAPK cascades, and transcriptional activation. Differences in the timing and levels of these signals influence the speed and strength at which these immune reactions are established and thus their effectiveness in counteracting the pathogens [4,8,9]. The plant hormones salicylic acid (SA) and jasmonic acid (JA) have been shown to play central modulating roles in this context. The activation of the JA pathway generally involves attacks from herbivores and necrotrophic pathogens. In grapevine cell cultures, JA and its bioactive conjugate jasmonoyl–isoleucine (JA-Ile) accumulate rapidly during PTI triggered by flg22, but not in cell death-related ETI induced by harpin [10]. In contrast, SA biosynthesis has mainly been observed to mediate resistance against biotrophic pathogens (i.e., under conditions where hypersensitive cell death occurs), in addition to its role in SAR [11,12].

Grapevine is among the crop plants with the highest economic yield per area; however, it is also among the crops with the highest infection rate from pathogens, such as downy mildew (*Plasmopara viticola*) and powdery mildew (*Erysiphe necator*). In North America, grape species have undergone coevolution with these pathogens, and as a result, they have developed specific ETI pathways involving different pathogen strains with different host specificities [13,14]. The European wild grape (*Vitis vinifera* ssp. *Sylvestris*), which is the ancestor of cultivated *V. vinifera* ssp. *vinifera*, does not exhibit ETI responses to downy mildew due to the lack of this coevolution, and both species are susceptible to downy mildew infection [15]. Rather, the tolerance of some *V. sylvestris* genotypes to pathogens, such as downy/powdery mildew and black rot (*Guignardia bidwelli*), is likely due to a more efficient PTI [16]. These pathogens were only introduced into Europe in the 19th century, and from there they spread to Central and East Asia. Consequently, ETI associated with these pathogens in wild Chinese grapevine species is not expected to be as widespread although its prevalence is not clear. Indeed, there is currently ongoing debate regarding the existence of ETI in non-American grapes, such as Chinese grapes [17]. Regardless, there are likely valuable wild grape genotypes exhibiting resistance to powdery/downy mildew [18].

One of the defense mechanisms employed by plants to inhibit bacterial and fungal growth is the production of stilbenes, which are also classified as phytoalexins. In grapevine, the main stilbenes are resveratrol and viniferin, which accumulate as a result of infection or stress and have been shown to efficiently block infection by pathogens such as downy and powdery mildew [19,20]. In a previous study, it was shown that of *V. rupestris* and *V. vinifera* cv. Pinot Noir cell cultures exhibit different patterns of stilbene production when induced by either flg22 or harpin [4].

R2R3-MYB transcription factors (TFs) represent a family of proteins that include the conserved MYB DNA-binding domain, which identified 134 members in grapevine until now [21]. A composite network consisting of overlaying maps of coexpression between structural and transcription factor genes, including MYB TFs, has been constructed and applied to search for berry-specific regulators of the phenylpropanoid pathway in grapevine [22]. MYB proteins are involved in the regulation of secondary metabolism output and signal transduction processes, such as stilbene synthesis, anthocyanin synthesis, flavonoid synthesis, lignin synthesis, and antistress signaling [23,24,25]. The grapevine MYB TFs *MYB5a*, *MYB5b*, *MYBPA1,* and *MYBF1* are found to modulate several branches of the flavonoid pathway [26,27,28]. Overexpression of *MYBF1* upregulates the genes related to flavonoid biosynthesis and thus improves tolerance to abiotic stresses in plants [29]. In another study of a group of grapevine R2R3-MYB-type transcription factors, *MYB14* and *MYB15* are shown to activate the promoters of stilbene synthase/resveratrol synthase (*StSy*/*RS*), a key enzyme in stilbene production [23]. We previously reported that a specific *MYB14* allele from *V. sylvestris* Hoe29 results in improved PTI, which is consistent with the stilbene synthase expression of triggering with flg22 [30].

Some Chinese wild *Vitis* show tolerance to pathogens to some degree, such as *V. quinquangularis* [20], *V. piasezkii,* and *V. pseudoreticulata* [31,32], but the resistant mechanism is still not clear and has to be further interpreted, although it is the native habitat for these pathogens. In addition, this non-full resistance is observed in most Chinese wild *Vitis* genotypes, and its resistance ability is higher than those of *Vitis vinifera* but lower than those of American grapes. More importantly, Chinese wild *Vitis* show variation in disease resistance, not only among species, but also, to some degree, among genotypes [18]. For example, *V. amurensis* Shuangyou has susceptibility to powdery mildew and high susceptibility to downy mildew, but the genotype of *V. amurensis* Heilongjiang has the resistance to both pathogens. The list goes on: for example, the *V. piasezkii* Liuba-8 is harboring superior resistance to powdery/downy mildew as well; however, *V. piasezkii* Gansu-91 does not have a similar resistance [18]. Therefore, an important goal in our work is to characterize the disease resistance of each species/genotype on a case-by-case basis. In our previous work, we collected some Chinese wild *Vitis* according to its resistance to common pathogens [18,20,31] through a field sampling technique in China, including different species and the same species but different genotypes.

In this current study, we targeted a genotype of Chinese wild grape, *V. quinquangularis* (*V. quinquangularis*-Pingyi), and a common susceptible grapevine species, the European *V. vinifera* cv. Carignan cultivar. Specifically, we investigated the mechanisms of resistance and conducted a comparative analysis of early defense signaling involving transcription factor *MYB14* triggered by flg22 and harpin in *V. quinquangularis*-PY. This is a novel data to explore the defense mechanisms from grapevine cell cultures to real plants, and it is also the first data to use *MYB14* as a genetic marker to investigate the tolerance mechanism in Chinese wild *Vitis*.

## 2. Results

### 2.1. R2R3-MYB Transcription Factors Involved in Plant Defense

In this study, we got the promoter sequences (~2000 bp) of all R2R3-MYB transcription factors from NCBI (https://www.ncbi.nlm.nih.gov/) and predicted the functional elements of the MYB promoters through PlantCARE (http://bioinformatics.psb.ugent.be/webtools/plantcare/html/). We found that MYB promoters harbored many elements related to abiotic and biotic stresses. For example, TC-rich repeats, which are reported to be involved in plant defense and stress responsiveness [33,34]; the promoter of *VvMYBPA1* contains TC-rich repeats and can regulate flavonoid branching pathway to produce proanthocyanidins, which are potentially able to confer protection against various abiotic and biotic stresses [28,35]; WUN-motif was related to wound responsiveness [36]; the promoter of *VvMYBC2-L2* contains WUN-motif element, which plays an important negative regulatory role in anthocyanin biosynthesis [37]; *VvMYB14* and *VvMYB15* contain WUN-motif element, which can strongly coexpress with STSs under a range of stress and developmental conditions, which is in agreement with the specific activation of STS promoters by these TFs [38]. The detailed information on the *cis*-elements of 134 R2R3-MYB transcription factors related to plant resistance can be found in Supplementary Table S1.

### 2.2. Distribution of MYB14 in the Grapevine Chromosome and Sequence Analyses of VqMYB14

**Figure 1:**
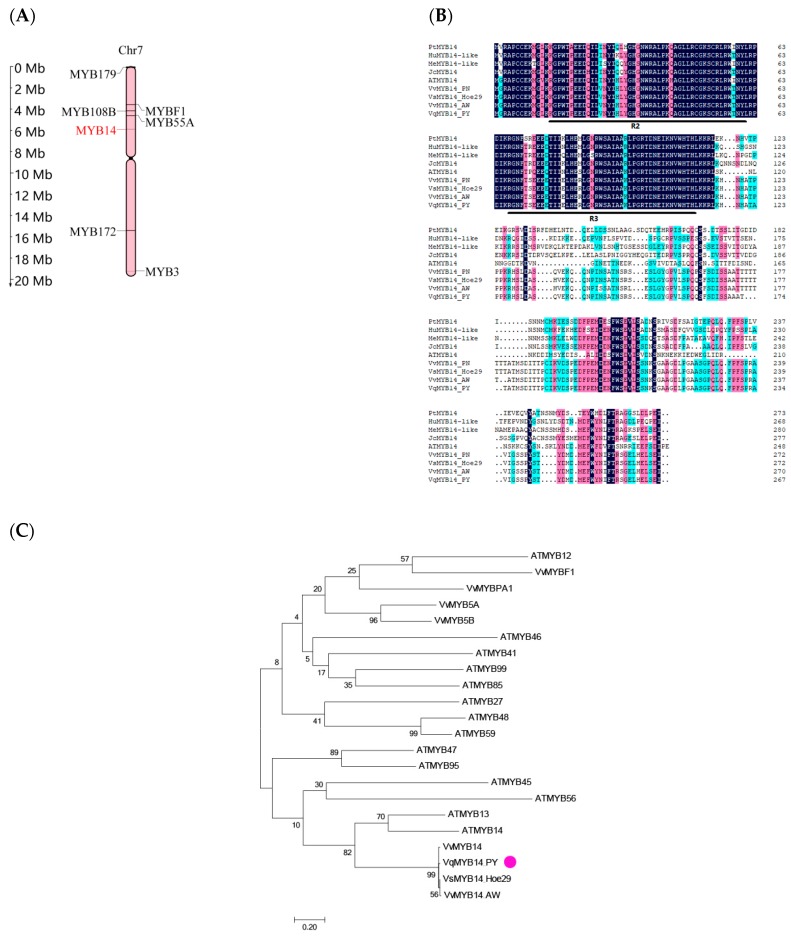
Physical location of *MYB14* in grapevine chromosome and putative peptide sequences of MYB14 proteins. (**A**) Mapping *MYB14* to physical location of grapevine chromosome 7 with respect to centromere positions. (**B**) The homology alignment of the putative amino acid sequence of VqMYB14_PY with that in other plants (PtMYB14, *Populus trichocarpa*, XP_002306849.2; HuMYB14-like, *Herrania umbratica*, XP_021279887.1; MeMYB14-like, *Manihot esculenta*, XP_021632420.1; JcMYB14, *Jatropha curcas*, XP_012085323.1) and grape varieties Pinot Noir (PN), Aguster Weiss (AW), and Hoe29 (*Vitis sylvestris*). The background color black indicates 100% homology, pink has 75% or more homology, and light blue has 50% or more homology. Black underline indicates that the feature is an R2MYB domain or an R3MYB domain. (**C**) Phylogenetic analysis of the relationships among VqMYB14_PY, other grape varieties (VvMYB14, Pinot Noir, NP_001268132.1; VvMYB14_AW, Aguster Weiss, a common *V. vinifera* cultivar; VsMYB14_ Hoe29, a *Vitis sylvestris* genotype) and MYB proteins in *Arabidopsis.* (The accession ID as follows: ATMYB45, At3g48920; ATMYB99, At5g62320; ATMYB85, At4g22680; AtMYB12, At2g47460; AtMYB13, At1g06180; AtMYB14, At2g31180; AtMYB27; At3g53200; AtMYB41, At4g28110; AtMYB46, At5g12870; ATMYB47, At1g18710; ATMYB48, At3g46130; AtMYB56, At5g17800; ATMYB59, At5g59780; ATMYB95, At1g74430; VvMYBF1, NP_001267930.1; VvMYBPA1, XP_010661716; VvMYB14, NP_001268132.1; VvMYB5A, NP_001268108.1; VvMYB5B, NP_001267854.1.)

Based on the classification of 134 R2R3-MYB family members and study of karyotype of grapevine [21,39], the distribution map of *MYB14* on the grapevine chromosome was made. We found that *MYB14* transcription factor was distributed on the short arm of chromosome 7 (Figure 1A). The *VqMYB14*_PY gene consists of 1303 bp with an 804 bp open reading frame (ORF) and encodes a putative protein of 267 amino acids with a predicted protein molecular weight of 29.9 kDa (SnapGene Viewer 4.2.11). VqMYB14_PY has two conserved MYB domains: an R2MYB domain between aa 14 and 61 and an R3MYB domain between aa 67 and 112 (Figure 1B). Phylogenetic tree results show that the sequence of VqMYB14_PY is highly similar to that of other grapevine varieties, and the similarity with *Arabidopsis* MYB14 is 52.22% (Figure 1C). All information for chromosome locations, cDNA, and protein sequences of R2R3-MYB family members can be found in Supplementary Table S2. 

### 2.3. Overexpression of VqMYB14 Enhanced the Stilbene Contents and Expression of Stilbene Biosynthesis Genes

**Figure 2:**
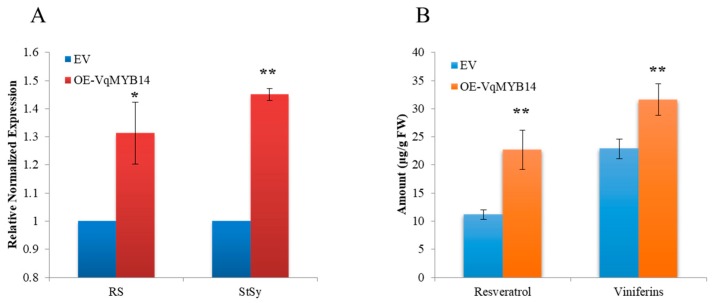
Overexpression of VqMYB14 and its effects on the main stilbenes accumulation in grapevine leaves. Leaves of *V. quinquangularis*-PY were infiltrated with *Agrobacterium* harboring the pCAMBIA1301 control and pCAMBIA1301-VqMYB14 overexpression constructs and were sampled after 36 h. (**A**) Real-time quantitative PCR analysis of *StSy* (X76892) and *RS* (AF274281) expression in leaves of VqMYB14-overexpressing (OE) and control (empty vector, EV). Expression levels of genes were normalized to *EF1-α* (EC959059) and were represented as expression relative to the EV value, which was set to 1. Primers of *StSy*/*RS*/*EF1-α* used for real-time quantitative PCR are listed in Supplementary Table S3. (**B**) Resveratrol and viniferins were extracted from EV and OE-VqMYB14 leaves and then quantified by high-performance liquid chromatography (HPLC) analysis (DaoJin LC-20A, Japan). The concentrations of resveratrol and viniferins in EV and OE-VqMYB14 leaves were calculated according to the characteristic peak area. * *p* < 0.05 and ** *p* < 0.01 indicate statistically significant differences. Data represent mean values from three independent experimental series. Error bars represent standard errors.

To investigate how VqMYB14 regulates stilbenes biosynthesis in grapevine, an agro-infiltration experiment was implemented in grapevine leaves of *V. quinquangularis*-PY. Because VvMYB14/VsMYB14 was shown to activate the promoters of stilbene synthase/resveratrol synthase (*StSy*/*RS*), a key enzyme in stilbene production [23,30], we investigated the mRNA expression of *StSy*/*RS* through overexpression of VqMYB14 in the leaves of *V. quinquangularis*-PY. As shown in Figure 2A, the expression of *StSy*/*RS* was significantly increased by the expression of VqMYB14 under the control of the CaMV 35S promoter at 36 h postinfiltration. Moreover, we detected the accumulation of *trans*-resveratrol and viniferins that were main antimicrobial stilbenes in infiltrated leaves. VqMYB14 overexpression led to significant increases of *trans*-resveratrol and viniferins compared with control leaves infiltrated with the empty vector (EV) (Figure 2B).

### 2.4. pVqMYB14 Activation is Stronger than pVvMYB14 Activation after flg22 and Harpin Induction

In addition to investigating the importance of VqMYB14 for stilbene accumulation in grapevine, we also monitored the potential response differences of the promoters of *MYB14* (*pVqMYB14*_PY and *pVvMYB14*_a common disease-susceptible *V. vinifera* cv. Carignan cultivar) to flg22 and harpin, which were well-characterized and often used experimentally to mimic certain aspects of PTI and ETI, respectively.

**Figure 3:**
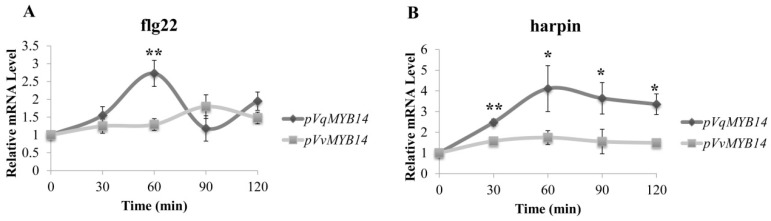
Activation of *pVqMYB14* and *pVvMYB14* by flg22 and harpin in a heterologous *Nicotiana benthamiana* expression system, determined using the GUS reporter. Values show promoter activities (GUS transcript abundance) relative to the untreated control after treatment with 1 μM flg22 (**A**) and 40 µg/mL harpin (**B**) at different time points (0 min, 30 min, 60 min 90 min, and 120 min). * *p* < 0.05 and ** *p* < 0.01 indicate statistically significant differences. Data represent mean values from three independent experimental series. Error bars represent standard errors.

The promoter activity was measured at different time points after treatment in a heterologous *Nicotiana benthamiana* system [40]. Approximately 30 min after flg22 treatment, *VqMYB14* promoter induction increased, peaking at approximately 60 min (2.7-fold). In comparison, the *VvMYB14* promoter induction was 1.2-fold at 60 min (Figure 3A). For harpin, *pVqMYB14* induction increased rapidly from about 30 min, peaked at about 60 min, and subsequently decreased slowly from 60 min to 120 min (Figure 3B). The *VqMYB14* promoter induction triggered by harpin was stronger (maximum of 4.1-fold around 60 min) than the response to flg22, whereas *pVvMYB14* showed no significant response. Taken together, the response of *pVqMYB14* was both faster and stronger than that of *pVvMYB14* after either flg22 (especially at 60 min) or harpin treatments (all expression points).

### 2.5. Differences in pVqMYB14 and pVvMYB14 Activities Depend on Structural Differences of the Promoters

**Figure 4:**
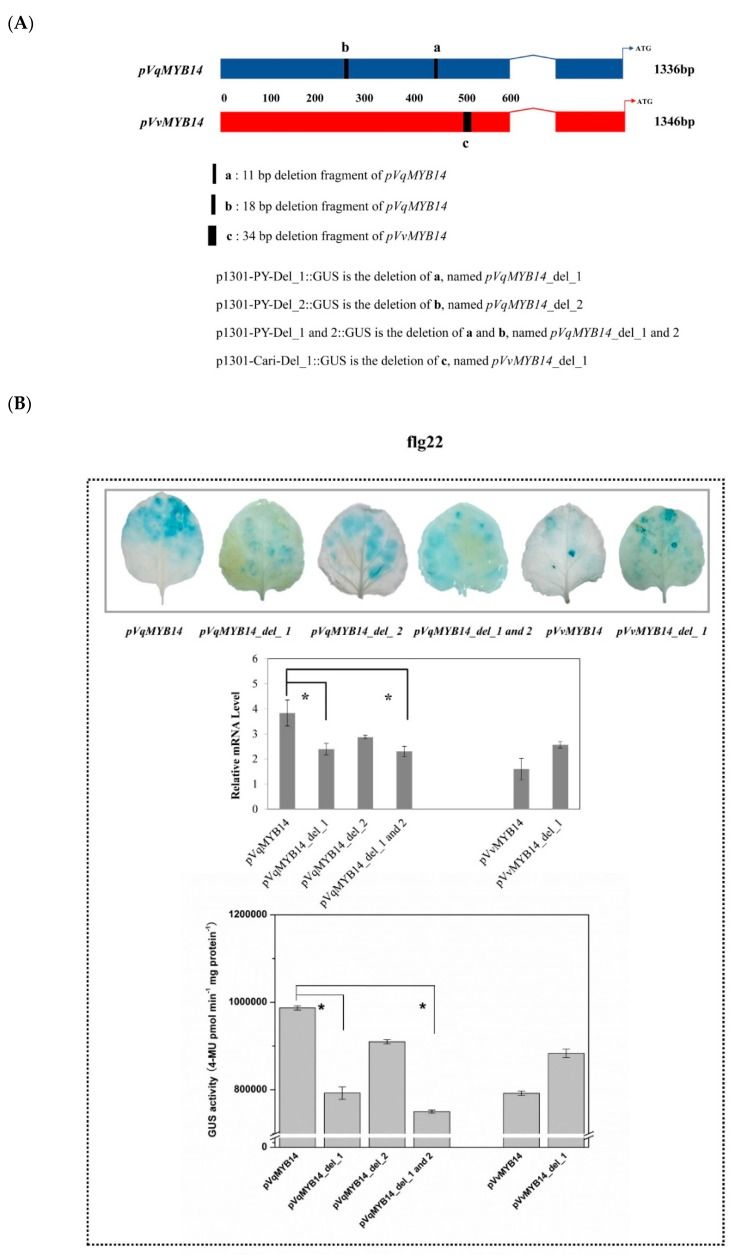

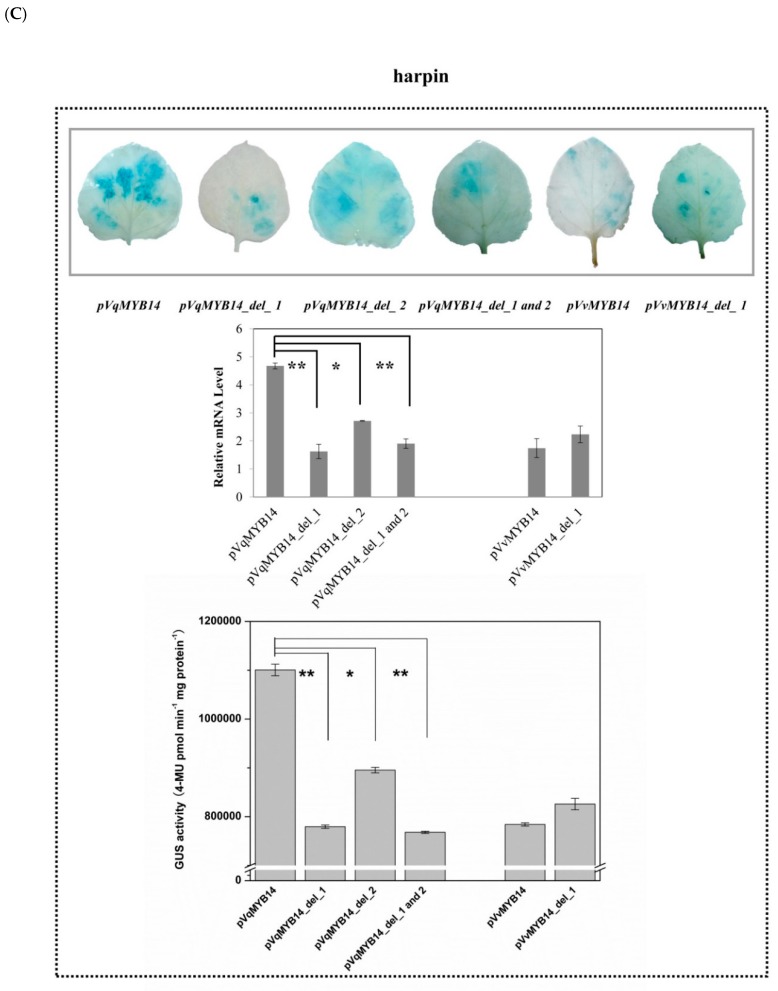
Comparison of the *MYB14* promoters from *V. quinquangularis*-PY and *V. vinifera* cv. Carignan. (**A**) Schematic diagram of the constructs highlighting the structure difference between *pVqMYB14* and *pVvMYB14*. Histochemical GUS expression assay, GUS transcript abundance based on real-time quantitative PCR (RT-qPCR), and GUS enzymatic activity in response to flg22 (**B**) and harpin (**C**) treatments for 1 h in transiently transformed *N. benthamiana* leaves. * *p* < 0.05 and ** *p* < 0.01 indicate statistically significant differences. Data represent mean values from three independent experimental series. Error bars represent standard errors.

To better understand the basis of the flg22/harpin-inducible *pVqMYB14* and *pVvMYB14* activities, we compared the promoter sequences. *pVqMYB14* and *pVvMYB14* are similar sizes (1,336 bp and 1,346 bp, respectively), but three distinct segments were found to differ between the two. *pVqMYB14* has two segments, ATATATTTATA (11 bp) and TAAATTTATTTTATTTAT (18 bp), that are missing from *pVvMYB14,* while *pVvMYB14* has a specific 34 bp sequence (AAAAATCAAAATATTTGATTTTTCTAATTTAACT) that is absent from the *pVqMYB14* promoter (Figure 4A). *pVqMYB14* and *pVvMYB14* were also analyzed using the PlantCARE algorithm [41] and PlantPAN 2.0 [42], and the *VqMYB14* promoter region was predicted to have more *cis*-elements than *pVvMYB14*, such as a TATA-box. Furthermore, *pVqMYB14* contains 25 more predicted transcription factor binding sites (TFBSs) than *pVvMYB14*. To test whether *pVqMYB14/pVvMYB14* expression induced by flg22/harpin correlates with these structural differences, we generated three *pVqMYB14* mutant constructs and one *pVvMYB14* construct. Activities of the constructs were tested using the GUS reporter after application of flg22 or harpin in a heterologous *N. benthamiana* system. Controls are shown in Supplementary Figure S1.

As shown in Figure 4B, the three *pVqMYB14* promoter constructs showed reduced induction compared with *pVqMYB14* following flg22 treatment, with two of them being substantial decreases in GUS activity and at the mRNA and protein levels. In contrast, no such increase was observed in *pVvMYB14* mutant. For the harpin-triggered inductions (Figure 4C), although all three *pVqMYB14* mutants had significantly reduced activities compared with *pVqMYB14*, one was much more reduced, with an mRNA induction of 1.6-fold rather than 4.7-fold. Again, the *pVvMYB14* mutant construct did not cause any significant increase or decrease.

These data suggest that the different activities of *pVqMYB14* and *pVvMYB14* induced by flg22 or harpin depend on the structural differences in the promoter regions. In this regard, the 11 bp segment in *pVqMYB14* seems to be particularly important for the induction by flg22/harpin.

### 2.6. Early Upstream Signaling Events Involved in flg22- and Harpin-Triggered Immunity of pVqMYB14

In order to identify which upstream signals might have been involved in the strong *pVqMYB14* induction, we characterized early signaling events, such as calcium influx, oxidative burst, MAPK cascades, and the JA and SA pathways [4,20] using *pVqMYB14* fused with a GUS reporter gene and transient expression in *N. benthamiana* leaves.

**Figure 5:**
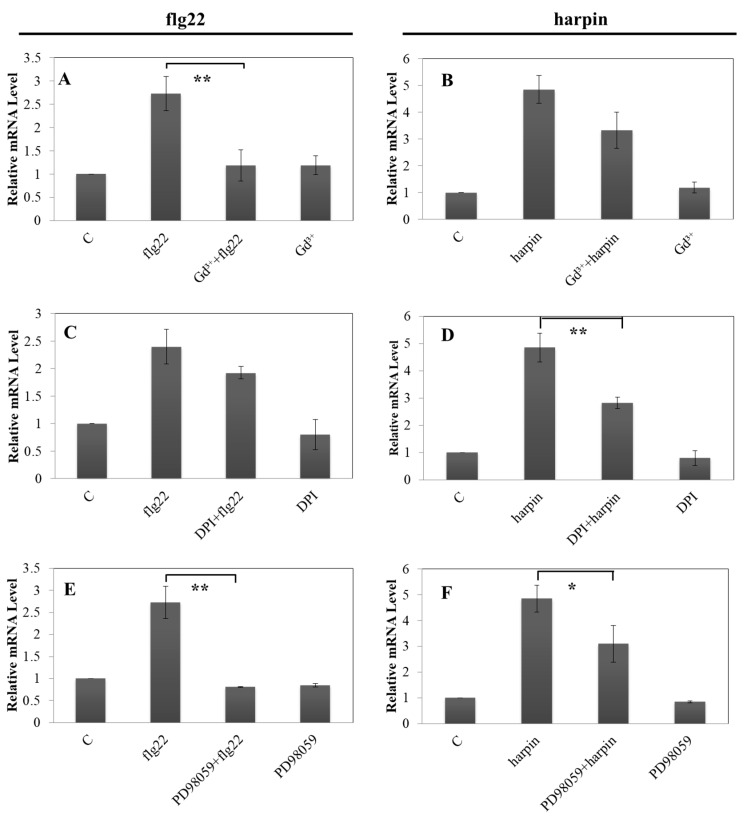
GUS transcript levels were measured by pretreatment with Gd^3+^/DPI/PD98059. GUS transcript levels resulting from the expression of *pVqMYB14::GUS* in response to flg22 and harpin treatments in combination with pretreatment with a 50 μM solution of the calcium-channel blocker Gd^3+^ (**A**,**B**), 100 μM solution of the NADPH oxidase inhibitor DPI (**C**,**D**), or a 100 μM solution of the MAPK cascade inhibitor PD98059 (**E**,**F**), as measured by RT-qPCR. GUS transcript levels were measured 1 h after the addition of flg22 or harpin, and pretreatments were for 30 min. * *p* < 0.05 and ** *p* < 0.01 indicate differences that are statistically significant. Mean values and standard errors are derived from three independent biological experiments.

The influx of Ca^2+^ is considered the earliest signaling event in plant immunity, so we used the calcium ionophore A23187 as a probe to permeate the cell membrane and release Ca^2+^ from outside of the cell membrane into the cytoplasm. This allows the calcium trigger to be sensed in the absence of an external stimulus. As shown in Supplementary Figure S2, this treatment induced promoter activity to around 2.4-fold compared to the untreated control for *VqMYB14*. The activity of a calcium influx channel is essential for the activation of early defense and can be blocked by GdCl_3_, an inhibitor of mechanosensitive calcium channels [43,44]. We therefore measured *pVqMYB14* promoter activity after flg22 and harpin induction in the presence of GdCl_3_. As shown in Figure 5A,B, when the plants were pretreated with GdCl_3_ for 30 min before the addition of flg22 or harpin, the activity decreased significantly from 2.7-fold, when flg22 alone was added, to 1.2-fold, which is close to control levels. No significant difference was seen for the harpin treatment system. These findings suggest that Ca^2+^ influx through the plasma membrane is required for the flg22/harpin induction of the *MYB14* promoter in *V. quinquangularis*-PY; however, the effect is more directly linked to the flg22-triggered response.

The rapid generation of reactive oxygen species (ROS), termed an oxidative burst, is an early inducible plant response during pathogen invasion or after treatment with elicitors [45]. To test whether the induction of *VqMYB14* requires an oxidative burst, the NADPH oxidase inhibitor, DPI, was used to quench the increase in ROS abundance following challenge with flg22 or harpin. As shown in Figure 5C,D, *VqMYB14* promoter induction was suppressed after pretreatment with DPI for both treatments; however, the degree of change was different. For the flg22-triggered response (Figure 5C), *pVqMYB14* induction decreased by approximately 30% when *pVqMYB14* was pretreated with DPI for 30 min. In contrast, *pVqMYB14* activity decreased by 53% during the harpin-triggered response (Figure 5D). Thus, the activation of *pVqMYB14* requires an oxidative burst for both flg22- and harpin-triggered activation, but it appears that ROS signaling is more important in the early stages of harpin induction than flg22 induction. 

The mitogen-activated protein kinase (MAPK) cascade represents one of the major signaling systems in eukaryotic cells, and we have shown it to be associated with the induction of plant defense responses [4,30]. To determine whether MAPK cascades are involved in *MYB14* promoter induction, a specific MAPK cascades inhibitor (PD98059) was used, and we observed that it blocked both flg22- and harpin-triggered *pVqMYB14* induction (Figure 5E,F).

### 2.7. The Role of SA in VqMYB14 Induction

**Figure 6:**
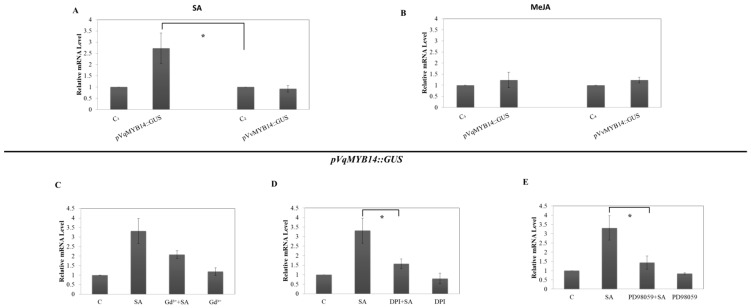
Activity of *pVqMYB14/pVvMYB14* in response to salicylic acid (SA) and methyl jasmonate (MeJA) in a heterologous *Nicotiana benthamiana* expression system, determined using the GUS reporter. Values show promoter activities (GUS transcript abundance) relative to the untreated control after treatment with 50 μM SA for 1 h (**A**) or 50 μM MeJA for 1 h (**B**). GUS transcript levels were measured 1 h after the addition of SA and the inhibitors Gd^3+^ (**C**), DPI (**D**), or PD98059 (**E**). Pretreatments were for 30 min. * indicates differences that are statistically significant (*p* < 0.05 level). Data represent mean values from three independent biological experimental series. Error bars represent standard errors.

The main plant hormones that regulate defense responses are salicylic acid (SA) and jasmonic acid (JA), and to assess their potential roles in *VqMYB14-* and *VvMYB14*-mediated signaling, we tested the responses of the respective promoters to SA and MeJA through expression of fusions with the GUS reporter gene in *N. benthamiana*. As shown in Figure 6A, SA activated *pVqMYB14* significantly, whereas *pVvMYB14* did not show any obvious induction. In contrast, both promoters responded similarly to MeJA treatment, with neither being activated (Figure 6B).

Since SA triggered a significant *pVqMYB14* response, we investigated the involvement of the same upstream signaling events as those associated with the flg22/harpin-induced responses. As shown in Figure 6C, pretreatment with GdCl_3_ influenced *VqMYB14* induction by SA but below the significance threshold. GdCl_3_ by itself did not cause any modulation of promoter activity. This result suggests that calcium influx acts as a positive modulator of the SA-dependent signaling activating the *VqMYB14* promoter.

To determine whether the activation of the *VqMYB14* promoter by SA is dependent on an apoplastic oxidative burst and the MAPK pathway, the inhibitors DPI and PD98059, respectively, were used. When the *VqMYB14* promoter was pretreated with either of these specific inhibitors for 30 min before SA treatment, there was a similar but stronger effect than for GdCl_3_ (Figure 6D,E). Again, these inhibitors alone did not produce any significant modulation. These results show that apoplastic ROS generated by NADPH oxidase and the MAPK pathway are necessary for the induction of the *VqMYB14* promoter triggered by SA.

### 2.8. flg22-Induced VqMYB14 Activation is More Sensitive to Gd Ions in Grapevine Than in a Heterologous Tobacco System

To further confirm that *VqMYB14* promoter activities induced by flg22 are differentially affected by Gd ions and DPI, we used a grapevine protoplast transient assay with a GFP reporter driven by the *VqMYB14* promoter (*pVqMYB14::GFP*). In agreement with previous observations (*pVqMYB14::GUS*, Figure 5A,C), flg22-induced GFP signals were weakened after the application of Gd ions and DPI, but the effect of the Gd ions was stronger (Figure S3B).

**Figure 7:**
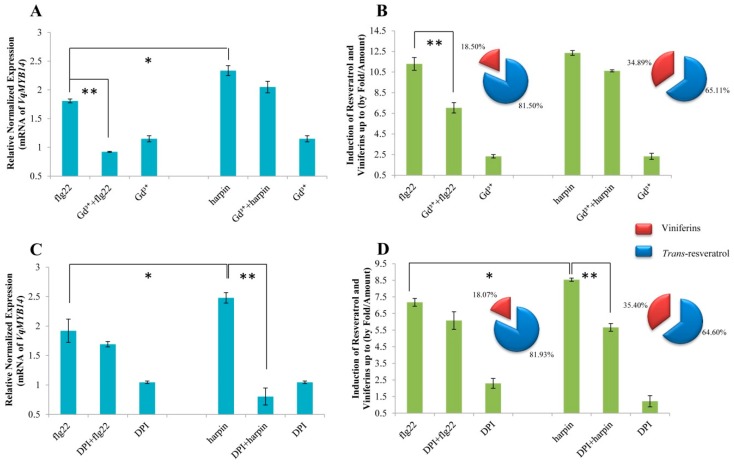
Expression analysis of *VqMYB14* and stilbenes levels in response to flg22 or harpin in the presence of Gd^3+^ or DPI. *MYB14* expression analysis, determined by real-time quantitative PCR (RT-qPCR) (**A**,**C**), and stilbene accumulation, including the relative proportion of *trans*-resveratrol versus viniferin, are shown (**B**,**D**) in the treated leaves of *V. quinquangularis*-PY. * *p* < 0.05 and ** *p* < 0.01 indicate differences that are statistically significant.. Data represent mean values from three independent biological experiments and error bars represent standard errors.

To further investigate whether the observed differences in induction of the promoter are correlated with the responses of *MYB14* in the leaves of *V. quinquangularis*-PY, we measured the transcript levels of *MYB14*, as well as stilbene accumulation. As shown in Figure 7A, when the grape leaves were pretreated with GdCl_3_ for 30 min before flg22 treatment, *MYB14* transcripts levels were significantly decreased, even to the level of the control, while the inhibition of NADPH oxidase by DPI had a similar but milder effect (Figure 7C). In the absence of flg22, the inhibitor alone did not affect the transcript levels. We also measured stilbene accumulation (*trans*-resveratrol and viniferins) after a GdCl_3_ (Figure 7B) or DPI (Figure 7D) pretreatment followed by flg22 treatment and found that stilbene accumulation decreased significantly in GdCl_3_-pretreated leaves compared with those that were DPI-pretreated. These results indicate that the *VqMYB14* response is more sensitive to calcium influx than other factors in flg22-triggered immunity.

### 2.9. Harpin-Induced VqMYB14 Activation is More Sensitive to DPI in Grapevine Than in a Heterologous Tobacco System

We repeated the experiment described above to investigate the effect on harpin-induced promoter activity. As expected, *VqMYB14* promoter-driven GFP was found to be strongly expressed in grapevine protoplasts treated with harpin (Figure S3C), and application of Gd ions and DP caused the harpin-induced GFP signals to be suppressed, with DPI having a stronger effect, as was observed with the GUS assays (Figure 5B,D).

We also investigated the *MYB14* expression levels, and as shown in Figure 7A,C, after treatment with harpin, we observed that they were suppressed by Gd^3+^ and DPI. However, this suppression was much stronger with DPI, which nearly abolished the *MYB14* induction caused by harpin. We also observed that the effect of Ca^2+^ and ROS on stilbene accumulation was different. DPI effectively suppressed the harpin-induced accumulation of stilbenes, while GdCl_3_ did not (Figure 7B,D).

In addition, although *V. quinquangularis*-PY accumulated the stilbene species included in both *trans*-resveratrol and viniferins after the elicitation of flg22/harpin, the proportions in total stilbenes are different. For flg22 induction, *trans*-resveratrol is the main stilbene species (~81–82%); in response to harpin, the ratio of *trans*-resveratrol remained ~65%, whereas viniferins made up ~35%.

In summary, *pVqMYB14* activity, *MYB14* expression levels, and stilbene accumulation were higher in harpin-induced than in flg22-triggered immunity, and accumulation of viniferins stilbenes was induced to a greater extent in harpin-triggered immunity. In addition, the effect of DPI was stronger in harpin treated samples and the role of Gd^3+^ was more significant in flg22 treated samples.

### 2.10. The Harpin-Induced Oxidative Burst Occurs Earlier Than flg22-Induced

**Figure 8:**
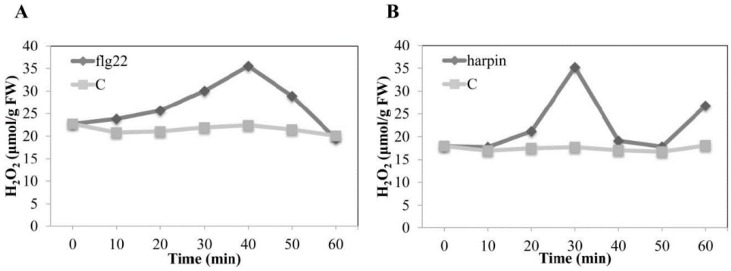
Production of hydrogen peroxide (H_2_O_2_). H_2_O_2_ was triggered by flg22 (**A**) and harpin (**B**) in the leaves of *V. quinquangularis*-PY. Leaves were incubated with sterile water for negative controls. Values represent means and standard errors from three independent biological replicates.

DPI effectively suppressed the harpin-induced accumulation of stilbenes, *MYB14* expression, and the induction of *pVqMYB14*, but it did not do the same during flg22-induced induction. To investigate why, and to test to what extent the oxidative burst is triggered by flg22 or harpin, we measured the H_2_O_2_ concentration in grape leaves at different time points. As shown in Figure 8, no significant changes were observed for the solvent control in the experiment, while the level of H_2_O_2_ was elevated after both flg22 and harpin treatments. As shown in Figure 8B, the signal increased immediately to a peak at 30 min after harpin treatment and then declined from 30–40 min. After this, there was another increase from 50–60 min. flg22-induced H_2_O_2_ production peaked at 40 min and then decreased from 40–60 min (Figure 8A). In summary, both flg22 and harpin induced an oxidative burst, but it occurred earlier in the harpin-treated samples, where it was also followed by a second wave of H_2_O_2_ production.

## 3. Discussion

China harbors many wild *Vitis* species [46,47,48], and some possess high or moderate pathogen tolerance [18]. Actually, Chinese wild grapevine is not expected to have a good disease resistance, because it is similar to European grapevine, has not co-evolved with the pathogens, and is not developing ETI against them [30]. Indeed, there is currently ongoing debate regarding the existence of ETI in non-American grapes, such as Chinese grapes [17]. For some European wild grapes (*V. vinifera* ssp. *Sylvestris*), their disease tolerance is likely to have a more efficient PTI [16]. Among these *V. sylvestris*, Hoe29 has even been identified as the reason for the improved basal immunity: it harbors a stretch in the control sequence of a gene switch (*MYB14*) that apparently was lost during domestication some thousands of years ago, and this specific region, in fact, can strongly promote the activation of this switch [20]. This switch, in turn, activates stilbene synthase, the key enzyme for the production of stilbenes [23] and could be a potential candidate target for resistance breeding in the future. In this study, we tried to use *MYB14* as a genetic marker to explore if the defense mechanism that exists in *V. sylvestris* also exists in wild grapes in China, or if they are involved in part of the resistance mechanisms that are present in wild grapes in Europe.

Actually, with our continuous in-depth research, we found the situation of resistance mechanism in Chinese wild grapes is more complicated because it shows variation in disease resistance, not only among species, but also, to some degree, among genotypes [18]. Therefore, the deep pathogen tolerance mechanisms in Chinese wild *Vitis* species are of great interest. 

**Figure 9:**
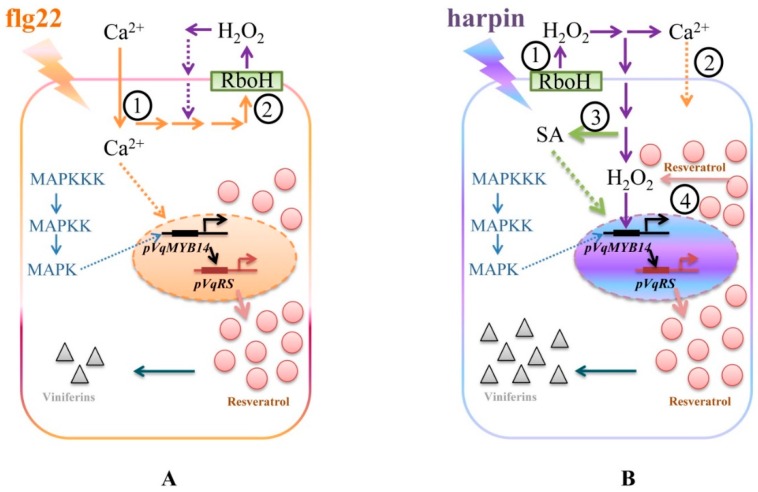
A model of upstream signaling in *V. quinquangularis*-PY. Model of flg22- (**A**) and harpin-induced (**B**) signaling events and stilbene profiles associated with *pVqMYB14* activity.

Plants react to different stress factors with specific responses. However, this specificity is determined by a limited number of signal molecules, such as Ca^2+^, ROS, and MAPKs, the effects of which may overlap. The perception of these common signals may be integrated and transduced in differing spatiotemporal patterns and contexts, triggering different immunity-associated responses (PTI versus ETI). In the current study, we used flg22 and harpin as representative elicitors to investigate the possible resistance mechanisms mediated by the transcription factor *MYB14* in Chinese wild *Vitis quinquangularis*-PY. A simple and experimental model describing the predictably associated defense processes based on this study is shown in Figure 9.

### 3.1. Calcium Influx

For flg22-induced immunity, we observed that calcium influx channels were more sensitive compared with harpin-triggered immunity (Figure 5, Figure S3, and Figure 7). In the previous work, by using cell cultures *Vitis rupestris* and the susceptible grapevine *Vitis vinifera* cultivar Pinot Noir, the flg22-induced alkalinization was more sensitive compared with the harpin-triggered response by GdCl_3_ inhibition, which indicates that flg22-receptor interacts more directly with calcium influx channels than the harpin–receptor complex, whereas ion influxes triggered by harpin might use other pathways [4]. Synchronously, in our experiments, we found that binding of flg22 to its receptor and calcium influx can be effectively inhibited by GdCl_3_, and the result is consistent with the previous observations by using cell cultures [4] and predicts that this process will result within a few minutes, in a substantial increase in cytosolic calcium levels in plant cells (Figure 9A①).

### 3.2. Oxidative Burst

Oxidative bursts have a dual function in defense systems: as early stress signals or as part of the downstream machinery that directly attacks invading pathogens [49]. flg22 activates membrane-bound NDAPH oxidase RboH via specific Ca^2+^-induced calcium-dependent protein kinases CDPKs [50], or by direct binding to its EF-hand motifs [51]. In addition, harpin could trigger an early oxidative-burst-preceded alkalinization, whereas this process was reversed in flg22 [4]. Therefore, the oxidative burst induced by flg22 binding does not act as an early signal, but rather represents a downstream response and occurs after the Ca^2+^ burst (Figure 9A②). Moreover, we observed a distinct difference in the induction of *pVqMYB14/MYB14* triggered by flg22 and harpin in the presence of the DPI inhibitor (Figure 5, Figure S3, and Figure 7). The activities of *pVqMYB14* and the transcript abundance of *MYB14* were significantly suppressed by the application of DPI in harpin-triggered immunity, but not in response to flg22. More importantly, we observed that the production of hydrogen peroxide (H_2_O_2_) (Figure 8), one of the major ROS species, showed differences in timing and extent after the treatment of flg22 or harpin. The oxidative burst was triggered by harpin at an early time point (0–30 min), but the effect flg22 was later (0–40 min). Thus, the oxidative burst seems to be employed as an early stress signal in response to harpin (Figure 9B①) but represents a downstream response in flg22-mediated responses (Figure 9A②).

### 3.3. MAPK Signaling

Many biotic and abiotic stresses activate MAPK cascades, which allow the translation of extracellular stimuli into intracellular responses such as interactions with transcription factors [52]. Here, we observed that PD98059, an inhibitor of MAPK pathways, blocked the flg22- and harpin-mediated induction of *pVqMYB14* (Figure 5E,F), thus showing that MAPK cascades are necessary for *pVqMYB14* activation in both responses.

### 3.4. SA Signaling

SA and JA play central roles in context PTI and ETI, respectively. However, the functions of phytohormones in defense are complex, and while the activation of the SA pathway is typically discussed in the context of cell-death-related defense [53], it is also involved in basal immunity [20]. Notably, flg22-induced basal immunity involves the accumulation of JA, but harpin does not [10]. We observed that SA activated *pVqMYB14*, but had no such effect on *pVvMYB14*, while MeJA induced neither of the promoters (Figure 6A,B). In addition to calcium influx, NADPH oxidase activity and the MAPK pathway were observed to be involved in SA-induced *pVqMYB14*. It was notable that the SA-mediated *pVqMYB14* induction was associated with the same signaling components as harpin and had a similar sensitivity to DPI, but not to Gd^3+^ (Figure 5B,D,F and Figure 6C,D,E). We propose that the oxidative burst is involved in early stress signaling associated with SA, consistent with the role of SA in ETI.

### 3.5. Stilbenes Output

In grapevine, the phytoalexins stilbenes accumulate as a result of pathogen infection or other stresses. Among the stilbenes, resveratrol and especially its oxidized products, viniferins, show particularly high toxicity and antimicrobial activity [54,55]. We observed that harpin induced *MYB14* transcripts and the accumulation of stilbenes, especially viniferins, to a greater degree than did flg22 (Figure 7). To summarize, flg22/harpin induces *MYB14*, which in turn activates stilbene synthase/resveratrol synthase to synthesis stilbenes. Resveratrol was reported to play a key role and amplifier of the oxidative burst in resistant *Vitis rupestris* that was absent in susceptible *Vitis vinifera* after the application of harpin [56], suggesting that harpin may promote a resveratrol-induced second wave of the oxidative burst in *V. quinquangularis*-PY (Figure 8B), and this may explain the differences in resveratrol and viniferin patterns in response to flg22 or harpin treatments (Figure 7). This result was also consistent with the previous studies in cell cultures [4]. In this study, although the total stilbene levels were higher in harpin treatment, the diversity is not so significant (Figure 7). The cause of this might be that all assays were performed on grapevine leaves, which were not as sensitive as grape cells. Besides, the treatment of flg22/harpin itself is too mild due to a low concentration of *trans*-resveratrol converts to other forms of stilbenes, such as piceid, piceatannol, or pterostilbene, which should be tested further. However, in combination with previous research reports and our solid experimental results, we still have a good explanation for all our experimental results.

In summary, we identified a transcription factor, *MYB14*, from Chinese wild grape, *V. quinquangularis*-PY, which can be proven to be very important for the accumulation of main stilbenes and expression of stilbene biosynthesis genes (*StSy*/*RS*). In addition, the application of Gd^3+^ and the decreases in *pVqMYB14* activities, *VqMYB14* expression, and resveratrol/viniferins accumulations were more significant in flg22-induced responses, while the effects of DPI were stronger following harpin elicitation. These findings suggest that upstream signaling in *V. quinquangularis*-PY is presented where early defense responses triggered by flg22 and harpin partially overlap, but where the timing and levels differ.

## 4. Materials and Methods

### 4.1. Plant Materials

Chinese wild *V. quinquangularis*-Pingyi (*V. quinquangularis*-PY) was collected in Pingyi County in the Shandong Province, China, and *V. vinifera* cv. Carignan was obtained from the Life Science Experimental Park at the Northwest University, Xi’an, Shaanxi, China. Tobacco plants (*N. benthamiana)* were grown in a growth chamber at 23 °C under long-day conditions (16 h light/8 h dark).

### 4.2. Gene Isolation and Sequence Analysis

Total RNA was extracted from grapevine leaves of Chinese wild *V. quinquangularis-*PY using an EZNA^®^ Plant RNA kit (Omega Bio-tech, Guangzhou, China) according to the manufacturer’s instructions. First-strand cDNA was synthesized using the *Evo M-MLV* RT-PCRT Kit (Accurate Biotechnology, Hunan, China). The cDNA of *VqMYB14* was amplified using Phanta Max Super-Fidelity DNA Polymerase (Vazyme, Nanjing, China) with gene-specific primers (Supplementary Table S4).

The homologous sequences of *VqMYB14*_PY were retrieved and analyzed using online BLASTP tools from NCBI (https://blast.ncbi.nlm.nih.gov/Blast.cgi). The sequences of AW (Aguster Weiss) and Hoe29 were obtained from [30]. The MYB protein sequences were aligned using DNAMMEN 9.0 version.

A phylogenetic tree of *VqMYB14*_PY, with its homologs in *Arabidopsis* and the *MYB14* sequences of different grapevine varieties Pinot Noir (PN), Aguster Weiss (AW), and Hoe29, was constructed using MEGA 7.0 with the maximum likelihood method (bootstraps  =  1000).

### 4.3. Overexpression of VqMYB14 in V. quinquangularis-PY

To identify the function of *VqMYB14*, the *VqMYB14* was cloned into the pCAMBIA1301 expression vector (Miaolingbio, Wuhan, China), and the GUS reporter gene of pCAMBIA1301 (http://www.miaolingbio.com/plasmid/P0277.html) was replaced with the *VqMYB14* using AhdI and BstEII. The overexpression of *VqMYB14* construct was introduced into *Agrobacterium tumefaciens* strain GV3101 (Weidi, Shanghai, China) by electroporation [57]. For *Agrobacterium,* mediated transient transformation in grapevine leaves were done as previously described [38], but with minor changes: the leaves from around 8 week in-vitro-grown *V. quinquangularis-*PY were selected and immersed in the *Agrobacterium* suspension; and the infiltrated leaves were obtained by vacuuming and maintained for 36 h under the growth chamber (23 °C, 16 h light/8 h dark), then washed with ddH_2_O three times, frozen immediately in liquid nitrogen, and kept at −80 °C until further use.

### 4.4. Plasmid Construction

Genomic DNA was isolated from leaves of the two grapevine genotypes using a plant genomic DNA extraction kit (BioTeke, Beijing, China) according to the manufacturer’s instructions. The full-length *MYB14,* including promoter and coding sequences, was amplified using the *MYB14-F/MYB14-R* primers (Supplementary Table S1), with *V. quinquangularis*-PY and *V. vinifera* cv. Carignan genomic DNA as templates. The PCR products were separately ligated into the T-Vector pMD™ 19 (Simple) vector (TaKaRa, Dalian, China) and transformed into *Escherichia coli* DH5α competent cells (TIANGEN, Beijing, China). The pMD-*VqMYB14* and pMD-*VvMYB14* plasmids were extracted from the resulting bacterial cultures using the TIANprep Mini Plasmid Kit (TIANGEN, Beijing, China) according to the manufacturer’s protocol.

The primers *pMYB14-Hind*III-F and *pMYB14-Bg*lII-R (Supplementary Table S1) were used to amplify the *MYB14* promoter fragments from the two grapevine genotypes using pMD*-VqMYB14* and pMD*-VvMYB14* as templates. The respective promoter sequences were ligated into the T-vector to obtain pMD*-pVqMYB14* and pMD*-pVvMYB14* and sequenced. Potential *cis*-elements and transcription factor binding sites in the promoter sequences were predicted with the online software packages Plant CARE (http://bioinformatics.psb.ugent.be/webtools/plantcare/html/) and PlantPan 2.0 (http://plantpan2.itps.ncku.edu.tw/promoter.php).

pMD*-pVqMYB14* and pMD*-pVvMYB14* were used as templates to amplify the *MYB14* promoter fragments with the primers PY_Del_1-F/PY_Del_1-R, PY_Del_2-F/PY_Del_2-R and Cari_Del-F/Cari_Del-R (Supplementary Table S1). The PCR fragments were ligated into a T-vector to generate pMD-*pVqMYB14_Del_1*, pMD-*pVqMYB14_Del_2*, pMD-*pVqMYB14_Del_1* and *2*, and pMD-*pVvMYB14_Del_1*. The T-vectors were then digested with the restriction enzymes *Hind*III and *Bgl*II and the fragments inserted into the pCAMBIA1301 expression vector (Miaolingbio, Wuhan, China) upstream of the β-glucuronidase (GUS) reporter sequence to generate *pVqMYB14::GUS*, *pVvMYB14::GUS*, *pVqMYB14_Del_1::GUS*, *pVqMYB14_Del_2::GUS*, *pVqMYB14_Del_1,* and *2::GUS* and *pVvMYB14_Del_1::GUS*.

The green fluorescent protein (GFP) sequence was amplified from the vector containing a GFP (Miaolingbio, Wuhan, China) fragment [58] using the GFP-F/GFP-R primers (Supplementary Table S1). The *GUS* fragment was removed from the *CaMV35S::GUS* and *pVqMYB14::GUS* using restriction enzymes *BstE*II and *Ahd*I, and the *GFP* fragment was inserted between the same restriction enzyme sites to generate plasmid *CaMV35S::GFP* and *pVqMYB14::GFP.*

### 4.5. Transient Expression

GUS expression vectors were introduced into *Agrobacterium tumefaciens* strain GV3101 by electroporation [57]. The bacterial suspensions (OD600 = 0.6) were then infiltrated into leaves of 6- 8-week-old *N. benthamiana* plants using a needle-free syringe [30]. After 48 h, the tobacco leaves were subjected to various treatments before assaying the GUS activity. GUS expression and GUS enzymatic activity were analyzed as described by [40] and [20]. The GFP expression vector was transformed into grapevine protoplasts using polyethylene glycol (PEG) as described in [59] and [60]. Protoplast isolation was performed according to [60], and cells were diluted to 2 × 10^5^ protoplasts mL^−1^ prior to various treatments. Fluorescence was observed by fluorescent microscopy (DMI3000B, Leica, Germany). The excitation wavelength of green fluorescent protein was 488 nm.

### 4.6. Treatment of Tobacco Leaves and Grapevine Protoplasts for Transient Promoter Assays

Tobacco leaves to be treated with different reagents were separately placed on filter paper in Petri dishes. For the induction treatments, the bacterial peptide flg22 (GenScript, Nanjing, China), the bacterial elicitor harpin (Lvyuan, Zhengzhou, China), SA (Solarbio, Beijing, China), and methyl jasmonate (MeJA) (Biotopped, Beijing, China) were prepared as previously described [4,20]. Tobacco leaves transiently expressing *pVqMYB14* and *pVvMYB14* were treated with 1 μM flg22 [30] and 40 μg/mL harpin [61]. Leaves were collected at 0 min (no treatment), 30 min, 60 min, 90 min, and 120 min after treatment. Tobacco leaves transiently expressing *pVqMYB14* were treated with 1 mM SA, 1 mM MeJA, and 50 μM A23187 (calcium ionophore) (Sigma-Aldrich, Shanghai, China) and collected after 1 h. For the inhibition treatments, 100 μM 2-(2-amino-3-methoxyphenyl)-4H-1-benzopyran-4-one (PD98059) (Sigma-Aldrich, Shanghai, China), 100 μM diphenyleneiodonium chloride (DPI) (Sigma-Aldrich, Shanghai, China), and 50 μM gadolinium chloride (GdCl_3_) (Sigma-Aldrich, Shanghai, China) were used. Stock solutions were prepared as described by [30]. Tobacco leaves were pretreated with the respective inhibitors for 30 min before flg22/harpin or SA was applied. Samples were collected 1 h after the elicitor application. A partial inhibition experiment was also performed using grapevine protoplasts, which were pretreated with DPI (10 μM)/GdCl_3_ (20 μM) [30] for 30 min and then incubated with 1 μM flg22/harpin (9 μg/mL) [4] for 4 h. Controls treated with the maximal concentration of the solvent used were also performed.

Transiently expressing (*pVqMYB14_Del_1*, *pVqMYB14_Del_2*, *pVqMYB14_Del_1* and *2,* and *pVvMYB14_Del_1)* tobacco leaves were treated with 1 μM flg22 and 40 μg/mL harpin and collected after 1 h. Control leaves were treated with sterile water.

### 4.7. Determination of H_2_O_2_ and Stilbene Levels in Grapevine Leaves

Randomly selected third to fifth fully expanded leaves from the plant apex of PY and Carignan were used for determining H_2_O_2_ levels after treatment with 1 μM flg22 and 40 μg/mL Harpin. Samples were collected at 0 min (no treatment), 10 min, 20 min, 30 min, 40 min, 50 min, and 60 min. H_2_O_2_ levels in the leaves were determined as described by [30] using a peroxide assay kit (Comin biotechnology Co., ltd. Suzhou, China). For determination of stilbene levels, grapevine leaves were pretreated with DPI (100 μM)/GdCl_3_ (50 μM) for 30 min and then incubated with flg22 (1 μM)/harpin (40 μg/mL) for 2 h. Stilbenes were extracted as described by [62] and analyzed as described by [30].

### 4.8. cDNA Synthesis and Quantitative Real-Time PCR

Total RNA was isolated from grapevine and *N. benthamiana* leaves using an EZNA^®^ Total RNA kit (Omega Bio-tech, Guangzhou, China) following the manufacturer’s instructions. mRNA was transcribed into cDNA using Prime Script Reverse Transcriptase (TaKaRa, Dalian, China) and quantitative real-time (qRT)-PCR amplifications were performed in triplicate, as described in [40]. Information on gene-specific primers is given in Supplementary Table S2. *NbEF1-α* (for *N. benthamiana* leaves) [63] and *EF1-α* (for grapevine leaves) [62] were used as reference controls.

## Supplementary Materials

Supplementary materials can be found at https://www.mdpi.com/1422-0067/21/3/706/s1.
