# Supplementary material for: The Effect of Transcription Factor MYB14 on Defense Mechanisms in Vitis quinquangularis-Pingyi
Source: Int J Mol Sci. 2020 Jan 21;21(3):706. doi: 10.3390/ijms21030706 (PMC7036875; doi:10.3390/ijms21030706)
Supplement: Supplementary file 1 [file ijms-21-00706-s001.zip › ijms-695011 supplementary/supplementary Figures.docx]

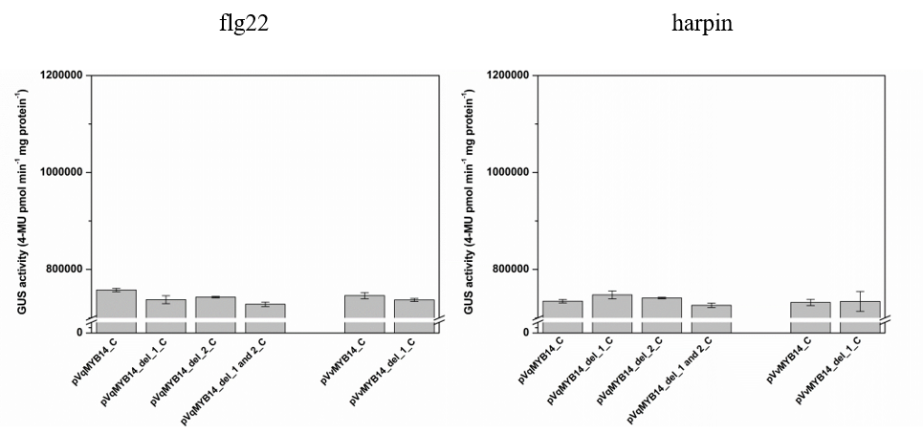


**Figure S1.** The negative controls of GUS enzymatic activity in response to flg22 and harpin treatments for 1 h in transiently transformed *N. benthamiana* leaves.


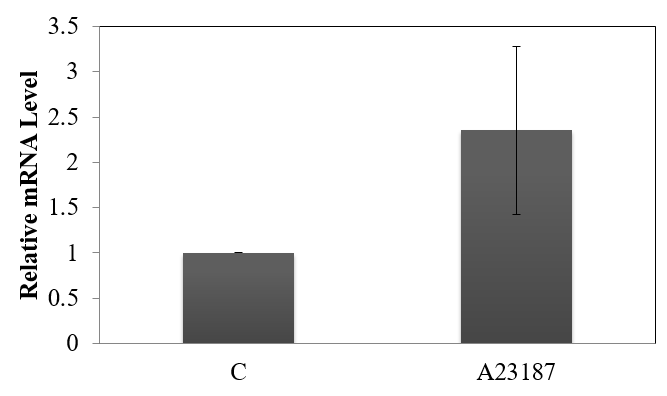


**Figure S2.** GUS transcript levels resulting from the expression of *pVqMYB14*::GUS in response to the calcium ionophore A23187. Values show promoter activities relative to the untreated control after treatment with 50 μM of A23187 for 1 h.

| (**A**)  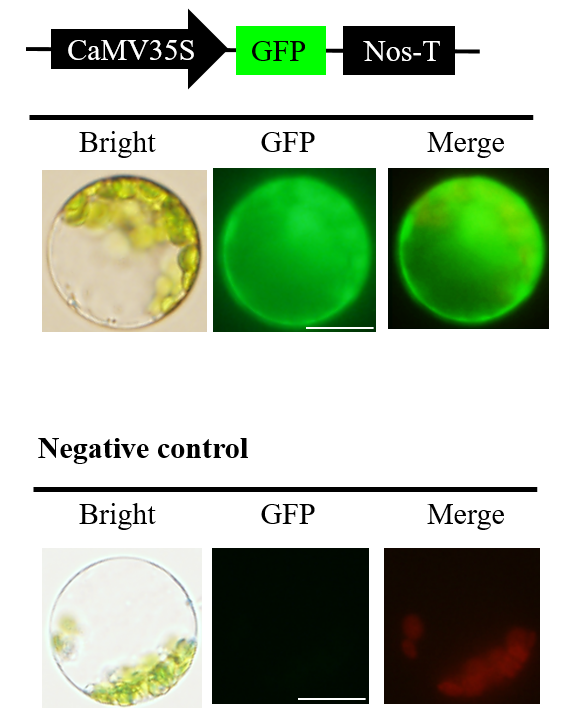 | (**B**)  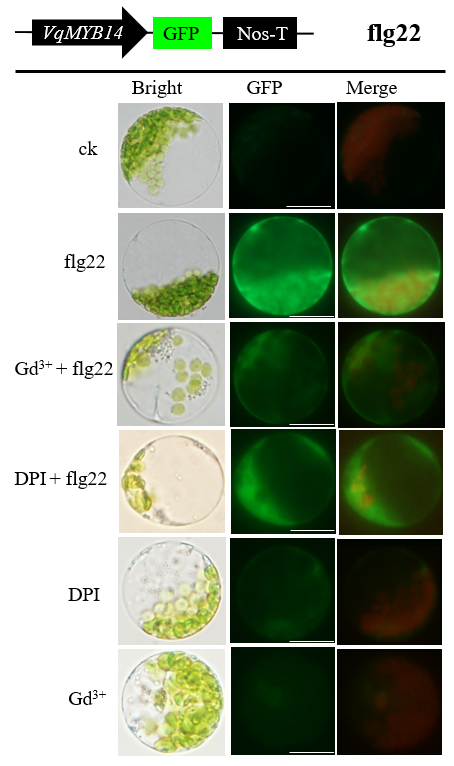 |
| --- | --- |
| (**C**)  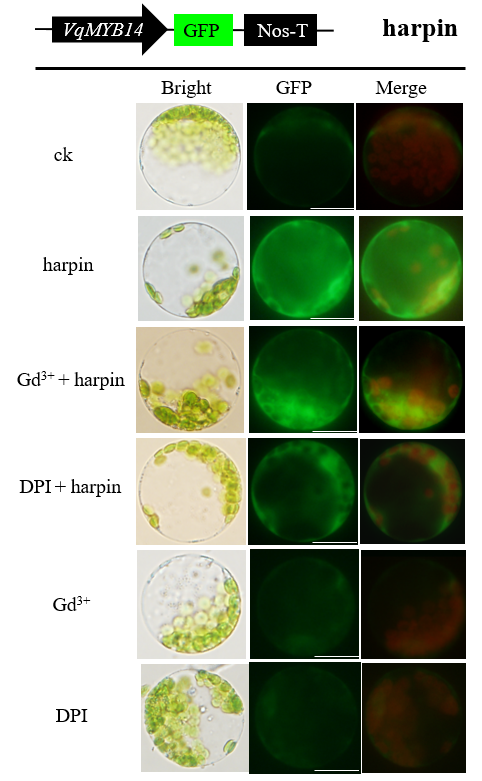 | |

**Figure S3.** Detection of *VqMYB14* promoter activities transiently expressed in *V. vinifera* cv. Cabernet Sauvignon protoplasts using a GFP assay, in response to flg22 or harpin in the presence of Gd^3+^ or DPI. GFP reporter constructs containing the *VqMYB14* promoter (*pVqMYB14*::GFP) and the CaMV 35S promoter (p35S::GFP; positive control) were transiently transfected into *V. vinifera* cv. Cabernet Sauvignon protoplasts using a polyethylene glycol (PEG) method and tested for *pVqMYB14* induction. Non-transfected protoplasts were used as a negative control. After incubation for 12 h, GFP fluorescence was observed. (**A**) Controls; (**B**) in response to flg22; (**C**) in response to harpin. Bars, 10 µm.
