# Supplementary material for: The Effect of Transcription Factor MYB14 on Defense Mechanisms in Vitis quinquangularis-Pingyi
Source: Int J Mol Sci. 2020 Jan 21;21(3):706. doi: 10.3390/ijms21030706 (PMC7036875; doi:10.3390/ijms21030706)
Supplement: Supplementary file 1 [file ijms-21-00706-s001.zip › ijms-695011 supplementary/Table S3.docx]

**Table S3.** Primers used for real-time quantitative PCR.

| **Name** | **GenBank Accession No.** | **Primer sequence 5'-3'** | **References** |
| --- | --- | --- | --- |
| *NbEF1-α* | AY206004.1 | Sense: 5’- -3’AGAGGCCCTCAGACAAAC  Antisense: 5’- -3’TAGGTCCAAAGGTCACAA | Zhang et al. (2015) |
| *GUS* | CP022959.1 | Sense: 5’- -3’ATTATGCGGGCAACGTCTGGTATCAG  Antisense: 5’- -3’CATCGGCTTCAAATGGCGTATAGC | Bai et al. (2019) |
| *EF1-α* | EC959059 | Sense: 5’- -3’TGTCATGTTGTGTCGTGTCCT  Antisense: 5’- -3’CCAAAATATCCGGAGTAAAAGA | Duan et al. (2015) |
| *MYB14* | ABW34392 | Sense: 5’- -3’GGGGTTGAAGAAAGGTCCAT  Antisense: 5’- -3’GGCCTCAGATAATTCGTCCA | Duan et al. (2016) |
| *RS* | AF274281 | Sense: 5’- -3’TGGAAGCAACTAGGCATGTG  Antisense: 5’- -3’GTGGCTTTTTCCCCCTTTAG | Duan et al. (2015) |
| *StSy* | X76892 | Sense: 5’- -3’ CCCAATGTGCCCACTTTAAT  Antisense: 5’- -3’ CTGGGTGAGCAATCCAAAAT | Duan et al. (2015) |
