# Supplementary material for: The Effect of Transcription Factor MYB14 on Defense Mechanisms in Vitis quinquangularis-Pingyi
Source: Int J Mol Sci. 2020 Jan 21;21(3):706. doi: 10.3390/ijms21030706 (PMC7036875; doi:10.3390/ijms21030706)
Supplement: Supplementary file 1 [file ijms-21-00706-s001.zip › ijms-695011 supplementary/Table S4.docx]

| *MYB14*-F | 5’ -CTACTGACGTGCACTAGCCT- 3’ |
| --- | --- |
| *MYB14*-R | 5’ -GCAGAGTGAAAGTGCAACACG- 3’ |
| *pMYB14*-*Hind*Ⅲ-F | 5’ -*AAGCTT*CTACTGACGTGCACTAGCCT- 3’ |
| *pMYB14*-*Bgl*Ⅱ-R | 5’ -*AGATCT*TTTTTCTTTTCTATGTAAGGATTTGAGACT- 3’ |
| *PY-Del-1*-F | 5’ -TTTATTCTTTAAAAATTAAAATTTATATATATAAAGGATGTAACCAATAAAATTTGA- 3’ |
| PY- Del-1-R | 5’ -CATATTTCAAATTTTATTGGTTACATCCTTTATATATATAAATTTTAATTTTTAAA- 3’ |
| PY- Del-2-F | 5’ -TTTCAAATTTCATCCATTAGTACTTTTATAAATTTATTTTTTTAAAGAGATTTAAATG- 3’ |
| PY- Del-2-R | 5’ -ATTTTCATTTAAATCTCTTTAAAAAAATAAATTTATAAAAGTACTAATGGATGAAATT- 3’ |
| *Cari*-Del-F | 5’ -TAGGGTGGTGTTTATTTTTTGATTGAATAGAAAAAATAACTTGTTCACATCGTTCAAT- 3’ |
| *Cari*-Del-R | 5’ -TTATATTGAACGATGTGAACAAGTTATTTTTTCTATTCAATCAAAAAATAAACACCAC- 3’ |
| *GFP*-F | 5’ -*GACATATCGTC*ATGGTGAGCAAGGGCGAG- 3’ |
| *GFP*-R | 5’ -*GGGTAACCC*CTTGTACAGCTCGTCCATG- 3’ |
| *MYB14C*-F | 5’-GACATGCCGTCATGGGGAGAGCTCCTTGTTGTGAGA- 3’ |
| *MYB14C-*R | 5’-GGTAACCTCATATTTCTGATAATTCATGCAACTCCCCG- 3’ |

**Table S4.** Primers used for constructing expression vectors.
